# External Beam Radiation Therapy for Intrahepatic Cholangiocarcinoma

**DOI:** 10.1007/s12029-026-01463-5

**Published:** 2026-04-17

**Authors:** Alexandra E. Hotca, Mary Feng

**Affiliations:** Department of Radiation Oncology, University of California San Francisco, San Francisco, CA USA

## Abstract

Intrahepatic cholangiocarcinoma (iCCA) is an aggressive primary liver malignancy with rising incidence and poor outcomes. Most patients present with unresectable disease, with mortality frequently driven by intrahepatic tumor burden and liver failure, making local control (LC) a central treatment goal. External beam radiation therapy (EBRT) plays an expanding role in multidisciplinary care, enabled by modern planning, image guidance, and motion management techniques that permit safe dose escalation. Across definitive EBRT series, a consistent dose–response relationship is observed. Higher biologically effective doses (BED) have been associated with improved outcomes, with BED > 80.5 Gy achieving superior 3-year LC and overall survival (OS) compared with lower-dose regimens. Dose-escalated approaches have reported 2-year LC rates of 80–90% and median OS approaching 20–30 months in selected patients. Advanced technologies such as proton beam therapy and MR-guided radiotherapy may further improve the therapeutic ratio, with reported grade 3 or higher toxicity rates below 8% and one- to two-year LC rates exceeding 90% in PBT series. Beyond definitive therapy, EBRT contributes to tumor downstaging, enabling surgical resection in approximately 10–30% of initially unresectable patients, and may improve survival in selected metastatic or liver-dominant disease. Early-phase data combining EBRT with immune checkpoint inhibition are emerging and warrant prospective validation.

## Introduction

Intrahepatic cholangiocarcinoma (iCCA) is a biologically aggressive malignancy arising from the intrahepatic biliary epithelium and represents the second most common primary liver cancer after hepatocellular carcinoma. Both incidence and disease-related mortality have increased globally and within the United States over recent decades [1]. Because iCCA is frequently diagnosed at an advanced stage, long-term outcomes remain poor, with reported five-year survival rates consistently below 20% [2]. Surgical resection offers the only potential for cure; however, the majority of patients present with disease that is unresectable at diagnosis. In this population, mortality is often driven by progressive intrahepatic tumor burden and hepatic failure rather than distant metastatic spread [3]. Given these disease characteristics, effective local and regional disease control represents a central therapeutic challenge in iCCA.

A variety of liver-directed treatment strategies have been employed in patients with unresectable disease, including thermal ablation, transarterial therapies such as transarterial chemoembolization (TACE) and selective internal radiation therapy (SIRT, also referred to as transarterial radioembolization or TARE), hepatic arterial infusion (HAI), and external beam radiation therapy (EBRT) [4]. These modalities have been applied in diverse clinical contexts, including definitive local therapy, tumor downstaging prior to resection or transplantation, and as adjuvant treatment following surgical resection. However, the comparative effectiveness of these approaches remains uncertain due to the absence of randomized trials and the predominance of retrospective series characterized by heterogeneous patient selection, tumor burden, and treatment techniques.

A systematic review of locoregional therapies highlights wide variability in reported outcomes and underscore the methodological limitations of the existing literature [5]. In this study, across modalities, median overall survival (OS) has been reported to range from the mid-teens to approximately 30 months. Improved outcomes associated with thermal ablation largely reflect selection of patients with small, focal tumors, whereas transarterial therapies have demonstrated more modest survival in broader populations. EBRT has consistently produced intermediate survival outcomes, with median OS approaching 19 months in heterogeneous cohorts, together with durable local control. However, direct comparisons across treatment modalities remain limited by substantial clinical and methodological heterogeneity.

Modern photon EBRT is typically delivered as an outpatient course over several treatment sessions, using daily image guidance (IGRT) to ensure accurate targeting of liver tumors despite respiratory motion and day-to-day anatomic variations. The process begins with a CT simulation, a dedicated planning visit in which the patient is positioned in a reproducible setup (often in a custom cradle or immobilization device) and a planning CT is acquired in the same position used for treatment. Respiratory motion management is central to liver SBRT, as reducing tumor movement allows for smaller treatment margins and more conformal, higher-dose delivery. Common approaches include active breath-holding, abdominal compression, respiratory gating, or real-time tracking, with technique selection guided by magnitude of tumor motion, patient tolerance and compliance, and available technology. The chosen motion management strategy is then incorporated into both planning and daily treatment delivery. Each treatment visit is non-invasive and painless, and usually takes approximately 15 to 30 min, with most of the time devoted to setup and imaging verification (commonly cone-beam CT with alignment to soft tissue and/or fiducials) prior to beam delivery. In select centers, proton therapy or MR-guided photon therapy may further improve normal tissue sparing and support online adaptive planning tailored to daily anatomy.

Advances in radiation planning, image guidance, and motion management have expanded the role of EBRT in iCCA by enabling safe dose escalation to complex hepatic targets while respecting normal liver and adjacent organ constraints. Unlike ablation or arterially directed therapies, EBRT is applicable across a broad spectrum of tumor sizes and locations, is independent of tumor vascularity, and remains feasible in clinical settings where biliary obstruction or altered hepatic perfusion may limit other liver-directed approaches. These attributes have supported the increasing integration of EBRT into multidisciplinary treatment strategies for iCCA.

This review summarizes the current evidence supporting the use of EBRT in iCCA, including its role in definitive treatment of unresectable disease, tumor downstaging prior to resection, investigational use as a bridge to liver transplantation, postoperative chemoradiation, metastatic consolidation, palliative symptom management, and emerging combinations with immunotherapy.

## Clinical Rationale for External Beam Radiation Therapy in Intrahepatic Cholangiocarcinoma

EBRT offers several practical advantages over other liver directed therapies that are particularly relevant in iCCA. EBRT can be applied to tumors across a broad range of sizes (even 15 cm or larger), whereas thermal ablation is generally limited to small, solitary tumors under 2–3 cm, restricting its applicability to a minority of patients [6]. EBRT can also safely target anatomically challenging lesions adjacent to major vessels, bile ducts, the gallbladder, and stomach or bowel, locations where thermal ablation may be less effective or high risk for complications.

Unlike arterially directed therapies, EBRT does not depend on tumor vascularity. iCCA is frequently hypovascular or heterogeneously vascular, which may limit the effectiveness of therapies reliant on selective arterial delivery. EBRT bypasses this limitation, allowing consistent dose delivery regardless of vascular characteristics. Eligibility considerations also differ across modalities. TARE is typically relatively contraindicated in patients with bilirubin levels exceeding 2 to 3 mg/dL depending on treated liver volume [7], a common clinical scenario in iCCA due to biliary obstruction. In contrast, EBRT may remain feasible when normal liver dose volume constraints can be respected, without strict absolute bilirubin thresholds. EBRT may also be considered in selected patients with limited extrahepatic disease, including treatment of distant oligometastases, whereas most arterial therapies are primarily applied in liver confined disease.

These practical advantages support broader clinical applicability of EBRT and may contribute to favorable outcomes across heterogeneous patient populations. Population based analyses, while subject to inherent selection bias, suggest that stereotactic body radiation therapy (SBRT) may be associated with improved OS compared with conventional chemoradiation or TARE in unresected iCCA cohorts [8]. Although such comparisons must be interpreted cautiously, they support the ability of EBRT to achieve meaningful disease control in clinical settings where other locoregional therapies may be limited.

Comparisons of toxicity across liver directed modalities are limited by the absence of randomized trials, but available data provide important context. A systematic review and meta-analysis of unresectable iCCA demonstrated that clinical adverse events occurred in 58.5% of patients treated with TACE compared with 43.0% following TARE, with a reported difference of 0.314, suggesting a higher toxicity burden with TACE [9]. Although CTCAE grade stratification was not provided, TACE is known to carry risks of post embolization syndrome, hepatic decompensation in patients with marginal liver reserve, and biliary complications, which may be particularly problematic in iCCA patients with preexisting biliary obstruction. For TARE specifically, the prospective observational CIRT registry study of 174 patients with unresectable iCCA reported 16% of grade 3 and 4 adverse events [10]. These included abdominal (2.9%), fatigue (2.9%), gastrointestinal ulceration (0.5%), gastritis (0.5%), radiation cholecystitis (0.5%), radioembolization induced liver disease (1.7%), and other events in 22 patients (12.6%). Although early experiences with SBRT with older technology similarly demonstrated meaningful rates of toxicity, advances in treatment planning and delivery have significantly improved treatment tolerance. In a phase I study evaluating SBRT for hepatocellular carcinoma and iCCA, the iCCA cohort experienced no grade 4 or 5 toxicities but a 30% rate of grade 3 toxicity (3 of 10 patients) [11]. These toxicities included radiation induced liver dysfunction, biliary strictures, and gastrointestinal mucosal injury, reflecting limitations of early treatment planning and dose constraints. With the adoption of modern radiation planning techniques, motion management, and refined organ at risk constraints, contemporary EBRT series demonstrate substantially improved safety profiles. In a recent cohort, grade 3 or higher gastrointestinal toxicity was reported in only 3.7% of patients [12]. Collectively, these data suggest that advances in EBRT technique and patient selection have markedly improved its therapeutic ratio, supporting a favorable toxicity profile relative to other liver directed therapies while maintaining effective LC.

## Definitive External Beam Radiation Therapy and the Role of Dose Escalation for Unresectable Intrahepatic Cholangiocarcinoma

Across published EBRT series in unresectable iCCA, a consistent observation is the presence of a dose–response relationship, in which higher delivered radiation dose is associated with improved LC and, in selected populations, improved OS. Because EBRT is delivered using a range of dose and fractionation schedules which impact the ultimate biologic effect, it is important to understand that radiation dose is commonly compared using radiobiologic metrics rather than physical dose alone. Two widely used measures are the biologically effective dose (BED) and the equivalent dose in 2 Gy fractions (EQD2). BED incorporates both total dose and dose per fraction to estimate the biological effect of radiation on tumor tissue, whereas EQD2 expresses the delivered dose as an equivalent dose had treatment been given in conventional 2 Gy fractions, facilitating comparison across studies and treatment regimens.

Advances in radiation delivery, particularly the adoption of intensity modulated radiation therapy (IMRT), have enabled more precise dose escalation in iCCA. IMRT modulates radiation intensity across multiple beam angles, allowing highly conformal dose distributions that improve sparing of normal liver and adjacent organs at risk such as the stomach, small intestine, and colon. This technical precision has facilitated the safe delivery of higher radiation doses and laid the foundation for modern hypofractionated approaches, including stereotactic body radiation therapy (SBRT), in appropriately selected patients. Hypofractionation refers to the delivery of larger doses per fraction over fewer treatments, typically 10 to 15 fractions, whereas SBRT represents a more extreme form of hypofractionation delivered in 3 to 5 fractions, both using rigorous image guidance and motion management to optimize both the effectiveness and safety of therapy.

**Fig. 1:**
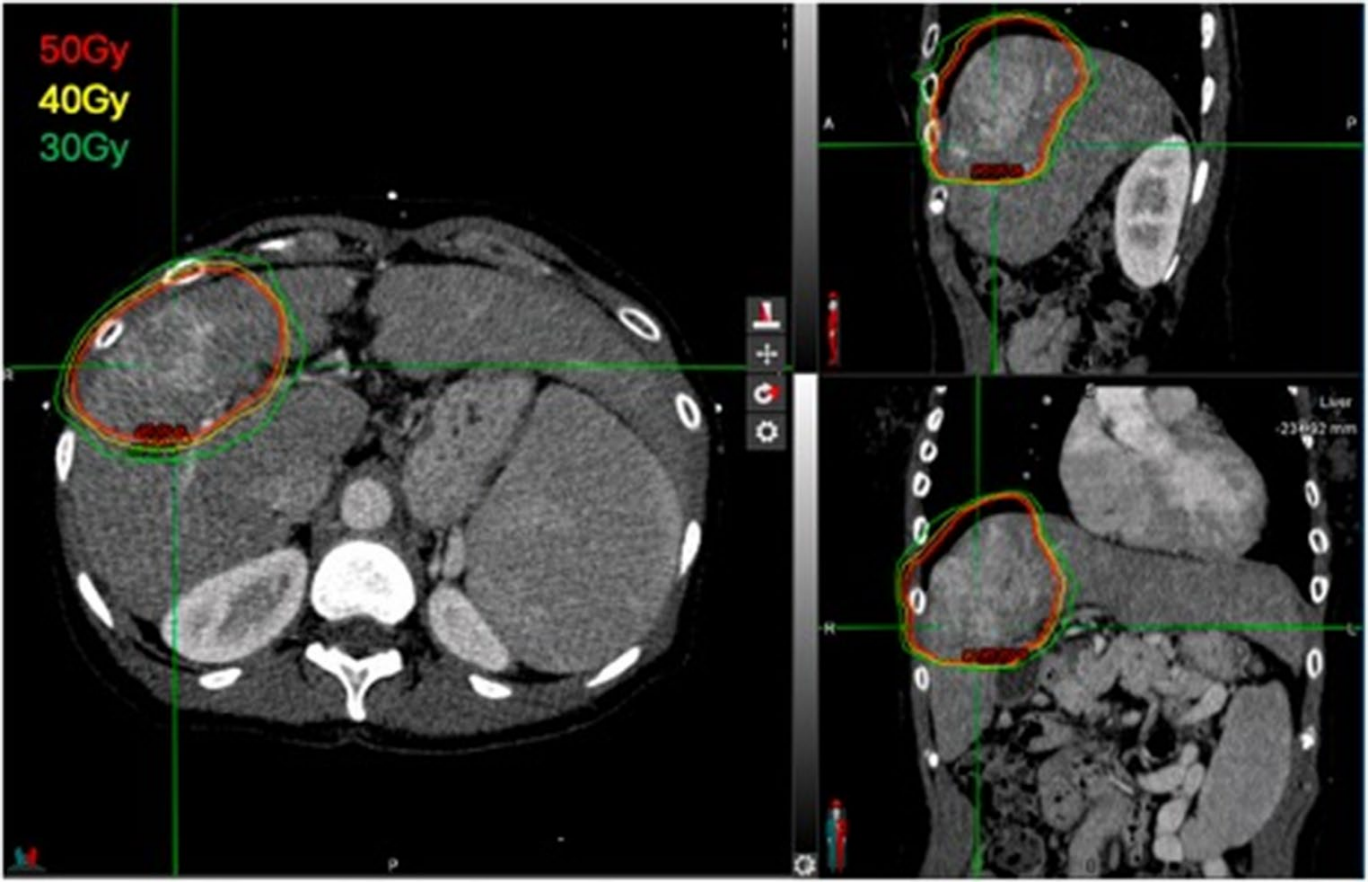
Representative SBRT plan for intrahepatic cholangiocarcinoma on the IV contrast planning CT simulation scan, shown in axial, sagittal, and coronal views. Isodose lines of 50, 40, and 30 Gy demonstrate conformal coverage of the arterially enhancing tumor and rapid dose fall off beyond the target to minimize irradiation of uninvolved liver and adjacent organs at risk. SBRT, stereotactic body radiotherapy; Gy, Gray

A representative SBRT plan for iCCA is shown in Fig. 1, illustrating conformal high dose radiation tightly shaped to the tumor with a uniform intratumoral dose distribution and a rapid dose fall off beyond the target to minimize exposure of surrounding normal liver and adjacent organs at risk.

Clinical outcomes with definitive EBRT using hypofractionation and SBRT have been favorable in selected patients. The American Association for the Study of Liver Diseases notes that SBRT can achieve median OS approaching 30 months in locally advanced disease, with higher radiation doses correlating with improved outcomes [13]. In a retrospective study of 66 patients treated with hypofractionated RT to a median dose of 58.05 Gy in 15 fractions, 2-year LC was 84% and 2-year OS was 58% [14]. Among patients treated with definitive rather than palliative intent, outcomes were further improved, with 2-year LC reaching 93% and 2-year OS 62%. Treatment was generally well tolerated, with grade 3 or higher toxicity reported in 11% of patients and one case of radiation induced liver disease.

Dose–response relationships have been further characterized in several analyses. In a cohort of 79 patients with inoperable iCCA, delivery of a BED greater than 80.5 Gy was associated with significantly improved outcomes compared with lower BED, including a 3-year OS of 73% versus 38% (*p* = 0.017) and a 3-year LC rate of 78% versus 45% (*p* = 0.04) [15]. Notably, the median tumor size in this cohort was 7.9 cm (range 2.2–17 cm), demonstrating the feasibility and efficacy of EBRT even in patients with large tumors. Additional analyses have demonstrated improved LC at higher EQD2, including a 1-year LC rate of 81.8% when EQD2 exceeded 71.3 Gy [16]. A recent meta-analysis of patients with unresectable or metastatic iCCA showed improved OS in patients treated with dose-escalated RT vs. conventional dose RT [12]. These findings are consistent with earlier observations that radiation dose escalation improves time to local progression in cholangiocarcinoma [17].

Several meta-analyses further support the use of SBRT in unresectable iCCA, although the available data remain largely retrospective. A 2025 meta-analysis of 13 studies including 366 patients treated with SBRT (mean dose 45 Gy), of whom the majority had iCCA (62.3%), reported a pooled median OS of 13.4 months, with pooled 1-year and 2-year OS rates of 58.7% and 33.2%, respectively, and pooled disease control rates of 84.7% at 1-year and 70.5% at 2-years [18]. Acute and late grade 3 + toxicity were reported in 6.4% and 16.4%, respectively. An earlier meta-analysis of 11 studies including 226 patients with unresectable or recurrent cholangiocarcinoma reported pooled 1-year LC of 78.6% and pooled 1-year OS of 53.8% following SBRT (median dose 45 Gy, range 30–55 Gy), with acute grade 3 or higher toxicity under 10% and late grade 3 or higher toxicity reported in 10–20% of patients [16].

Prospective evidence remains limited and highlights the importance of disease site specific interpretation. In the phase II ABC-07 multicenter randomized study, which enrolled patients with inoperable locally advanced cholangiocarcinoma across subsites including perihilar (46%), distal (39%), and intrahepatic disease (14%), the addition of SBRT following chemotherapy did not improve progression free survival and did not demonstrate an OS benefit [19]. While the heterogeneous subsite distribution and small proportion of iCCA patients limit disease specific inference, this trial underscores the need for refined patient selection and prospective studies focused specifically on intrahepatic disease, which can compromise liver function due to tumor bulk or biliary obstruction.

Current guideline recommendations support the use of dose escalated EBRT approaches when normal tissue constraints can be respected in the treatment of primary liver cancers. The American Society for Radiation Oncology (ASTRO) recommends hypofractionated regimens of 58 to 67.5 Gy in 15 fractions using photon or proton therapy [20]. When SBRT is feasible, doses of 40 to 60 Gy in 3 to 5 fractions are recommended, particularly for well selected tumors with adequate separation from critical organs at risk such as bowel. When neither hypofractionated dose escalation nor SBRT is feasible due to tumor size, location, or normal tissue constraints, conventional fractionation approaches remain appropriate, including 60 Gy in 30 fractions up to 77 Gy in 35 fractions, with or without concurrent chemotherapy.

## Modern Radiation Techniques Enabling Safe Delivery of High Dose Radiation: Proton Beam Therapy and MR Guided Radiotherapy

Advances in radiation technology have expanded the population of patients in whom ablative dose radiation can be delivered safely, particularly those with tumors adjacent to luminal gastrointestinal structures or those in whom preservation of uninvolved liver parenchyma is critical. In radiation oncology, an ablative dose generally refers to delivery of a very high biologic radiation dose, typically defined as a BED greater than 100 Gy, with the intent of achieving durable local tumor eradication analogous to surgical resection or thermal ablation. Achieving such dose levels in the liver is inherently challenging because of proximity to radiosensitive gastrointestinal organs, and limited normal liver reserve, making advanced delivery techniques particularly relevant in iCCA.

### Proton Beam Therapy

Proton beam therapy (PBT) differs from conventional photon-based radiation in its fundamental dose deposition characteristics. Protons deliver the majority of their energy at a relatively finite depth within tissue, producing a pronounced peak in dose known as the Bragg peak, followed by a rapid distal falloff. This physical property allows radiation dose to be conformed to the target volume while minimizing exit dose to surrounding normal tissues, in contrast to photon beams, which continue to deposit some dose beyond the target. With advanced treatment planning, safety is prioritized, and dose to each voxel of tissue in each organ is minimized as much as possible and kept below safe limits. In the stomach and bowel, most dose limits are related to maximum dose and not improved with PBT. However, in the liver, where integral dose determines overall tolerance, preservation of uninvolved parenchyma is critical, since this reduction in integral dose may offer a meaningful therapeutic advantage. By limiting unnecessary radiation exposure to normal liver, PBT may enable delivery of higher biologic doses, potentially increasing local control. PBT can be delivered using conventional fractionation, moderate hypofractionation, or SBRT, similar to photon-based EBRT. These characteristics make PBT potentially attractive for patients with large tumors, centrally located lesions, and especially in patients with limited liver reserve in whom photon-based dose escalation may be constrained. On the other hand, image guidance and motion management for are challenging for PBT.

Clinical evidence supporting PBT in cholangiocarcinoma continues to evolve. In a Japanese multicenter prospective registry study of high dose PBT for intrahepatic and perihilar CCA, 133 patients with iCCA were treated with a median dose of 80 Gy (relative biological effectiveness, RBE) in 25 fractions [21]. Commonly used regimens included 72.6 Gy (RBE) in 22 fractions and 76 Gy (RBE) in 20 fractions. Median OS for iCCA was 17 months, with 1-year and 2-year OS rates of 68.8% and 34.4%, respectively. Notably, among patients with tumors located at least 2 cm from the gastrointestinal tract who received higher dose distributions corresponding to an EQD2 of at least 74 Gy, the combined intrahepatic and perihilar cohort achieved a median OS of 37 months and a 2-year OS of 61.8%. Grade 3 or higher adverse events occurred in 5.3% of patients, most commonly bile duct stenosis, with additional events including gastric bleeding, duodenal perforation, and cholangitis.

In a multi-institutional nonrandomized study of patients with primary liver cancers, 39 patients with unresectable iCCA were treated with hypofractionated proton therapy (median dose of 58 Gy (RBE) in 15 fractions) [22]. For iCCA cohort they reported a 2-year OS of 46.5% and 94.1% LC rate at 2-years. For iCCA patients grade 3 toxicity was 7.7% and no grade 4 or 5 toxicity were reported. Additionally, a multi-institutional prospective registry reported a 1-year LC rate of 90.9% and a 1-year OS of 81.8% for patients with iCCA treated with hypofractionated proton therapy to a median dose of 80.5 Gy (RBE) [23]. They reported grade 3 or higher toxicity rate of 4.8%, there was no Grade 5 toxicity and only one patient in the liver cancer cohort experienced one grade 4 hyperbilirubinemia. PBT can be delivered safely in patients with unresectable iCCA, with reported rates of grade 3 or higher toxicity below 8% and high LC, with one- and two-year LC rates above 90%.

### MR-guided Radiotherapy

Magnetic resonance-guided radiotherapy (MRgRT) and daily online adaptive radiotherapy represent additional technological advances designed to address key limitations of conventional CT-guided liver SBRT. While CT-based image guidance has enabled significant improvements in local control, its application in liver tumors remains challenging due to limited soft tissue contrast. Liver tumors are often poorly visualized on cone beam CT, necessitating surrogate localization strategies such as implanted fiducial markers or reliance on adjacent anatomy. In addition, CT-guided approaches without daily adaptive replanning offer limited ability to account for day-to-day variation in bowel position, which can be particularly consequential for tumors located near gastrointestinal organs at risk. In this situation, safety must be prioritized, so a portion of the tumor may be underdosed that day. MRgRT overcomes many of these limitations through the integration of MR imaging directly into the treatment delivery system. The superior soft tissue visualization afforded by MRI allows direct identification of liver tumors and adjacent gastrointestinal structures at the time of treatment, enabling smaller treatment margins and reducing reliance on invasive fiducial placement. A defining feature of MRgRT is the capacity for online adaptive radiotherapy, in which a new treatment plan is generated based on the patient’s anatomy on the day of treatment. This capability allows clinicians to respond in real time to changes in bowel position, organ motion, or liver deformation that might otherwise require compromising complete tumor treatment for the sake of safety.

Although clinical experience with MRgRT in iCCA remains limited due to the relative novelty, expense, and high staffing needs of the technology, early outcomes in liver tumors treated in anatomically challenging locations have been encouraging. These data suggest that MRgRT may expand the population of patients eligible for ablative-intent EBRT while maintaining acceptable toxicity profiles. In a phase I trial of MRgRT for liver tumors in challenging locations, which included patients with iCCA, local control was 100% at a median follow up of 15 months with no grade 3 or higher gastrointestinal toxicities reported [24]. In a multi-institutional retrospective study of MRgRT for 26 patients with liver tumors treated with stereotactic MRgRT, including two patients with CCA, to a median dose of 50 Gy in 5 fractions, showed that at a median follow up of 21.2 months, freedom from local progression in the cholangiocarcinoma patients was 83%, with pooled 1-year and 2-year OS rates of 69% and 60%, respectively [25]. Grade 3 gastrointestinal toxicity occurred in two patients, both of whom had received prior local liver directed therapies.

Together, PBT and MRgRT represent complementary strategies to improve the therapeutic ratio of EBRT in iCCA. By enabling safer delivery of high biologic doses in anatomically challenging settings, these technologies expand the subset of patients who may be candidates for ablative intent radiotherapy.

## External Beam Radiation Therapy for Tumor Downstaging and Conversion to Resection

Complete surgical resection remains the only potentially curative treatment for iCCA, making conversion of borderline resectable or unresectable disease to resection an important clinical objective. Although prospective data are limited, retrospective series suggest that chemoradiation (chemoRT) can enable curative resection in a subset of patients initially deemed unresectable, with postoperative outcomes comparable to those achieved with upfront surgery in carefully selected cohorts. In a retrospective study evaluating chemoRT for initially unresectable iCCA, 12.5% of patients ultimately underwent curative resection [26]. Patients who proceeded to surgery demonstrated significantly improved outcomes compared with those treated with chemoradiotherapy alone, including a 3-year local recurrence free survival of 50% versus 15.7% (*p* = 0.03), and a 3-year OS of 50% versus 11.2% (*p* = 0.012). Factors associated with successful conversion included receipt of gemcitabine, delivery of higher radiation dose corresponding to a BED of at least 55 Gy, and a reduction in CA19-9 levels greater than 70%. Reported Grade 3 toxicity were anorexia (7.8%), nausea or vomiting (4.8%), and epigastric pain (10.9%). One patient developed gastric bleeding related to a grade 2 ulcer after receiving 40 Gy and was managed medically. Notably, no perioperative complications were reported among patients who underwent chemoradiotherapy followed by surgical resection.

In another neoadjuvant chemoRT series of 15 patients, including 7 with iCCA, radiographic tumor shrinkage was observed in 6 of 7 patients (85.7%) [27]. By RECIST criteria, 57.1% achieved a partial response, 14.3% had stable disease, and 28.6% experienced disease progression. Five of the seven iCCA patients were deemed resectable following chemoRT and subsequently underwent radical hepatectomy. Among these patients, severe surgery related complications occurred in 27.3%, including one patient with postoperative liver failure and one with pleural effusion, while one patient experienced severe gastritis attributed to radiotherapy. Ninety-day postoperative mortality was zero. Across patients who underwent resection, median OS was 37 months, with 1-year, 2-year, and 5-year survival rates of 80.8%, 70.7%, and 23.6%, respectively.

Taken together, these data support the consideration of chemoRT as part of conversion strategies for select patients with initially unresectable intrahepatic cholangiocarcinoma, while emphasizing the importance of careful multidisciplinary patient selection, particularly in the choice of chemoRT or multi-agent systemic therapy as neoadjuvant therapy, close monitoring of treatment response, and rigorous perioperative risk assessment.

## External Beam Therapy as a Bridge to Liver Transplantation

Although not common, liver transplant can be used for patients with cholangiocarcinoma. Transplantation strategies are best established for perihilar cholangiocarcinoma, in which neoadjuvant chemoradiation followed by liver transplantation has produced durable long-term survival, beginning with the Mayo Clinic approach [28]. Additional series have reported similarly favorable outcomes using neoadjuvant chemoradiation followed by transplantation in carefully selected patients [29–31].

For iCCA, transplantation remains investigational outside of clinical trials in most settings. Current guideline-based transplant exception considerations emphasize strict eligibility criteria for iCCA. Eligibility requires biopsy proven iCCA, underlying cirrhosis, unresectable disease, receipt of prior locoregional or systemic therapy, and demonstration of disease stability for at least six months from diagnosis or completion of treatment without new lesions or extrahepatic disease [32].

Retrospective analyses suggest that outcomes are most favorable for patients with very early-stage disease in the setting of cirrhosis. In one series, patients with small solitary tumors measuring up to 2 cm achieved OS rates of 100%, 73%, and 73% at 1, 3, and 5 years, respectively, whereas larger or multifocal tumors were associated with poorer outcomes [33]. Comparable survival has also been reported in selected patients with lesions up to 5 cm, with 1, 3, and 5 year overall survival rates of 90%, 76%, and 67%, respectively [34]. For patients with unresectable, locally advanced intrahepatic cholangiocarcinoma arising in non-cirrhotic livers without extrahepatic disease, prospective cohorts have explored transplantation following sustained disease stability on systemic therapy, with or without liver directed therapy, demonstrating promising outcomes in highly selected populations [35, 36].

Within this evolving landscape, EBRT has been incorporated into transplant bridging strategies in limited series. In a single center retrospective study of 26 patients, predominantly with perihilar disease but including a subset with intrahepatic tumors (15.4%), preoperative SBRT to a dose of 40 to 50 Gy in 5 fractions followed by consolidation chemotherapy prior to orthotopic liver transplantation was evaluated [37]. Among the nine patients who ultimately underwent transplantation, 3-year OS was 75%, compared with 9% among patients who dropped out due to disease progression. SBRT was generally well tolerated, with 30% of patients developed acute Grade 1 toxicity and one patient suffered a duodenal perforation three months after completion of SBRT.

Ongoing clinical trials, including NCT04195503, and contemporary analyses continue to refine transplant candidacy and treatment frameworks [38]. Overall, while EBRT appears feasible as part of transplant bridging strategies, its role in iCCA transplantation remains investigational, center dependent, and best pursued within prospective clinical trials or highly structured multidisciplinary protocols.

## Adjuvant Radiation Therapy after Surgical Resection

The role of adjuvant RT following resection of iCCA remains controversial. Available evidence is derived largely from retrospective analyses with heterogeneous patient populations, variable radiation techniques, and uncertainty regarding optimal target definition. As a result, reported outcomes have been inconsistent across datasets.

Early population-based analyses suggested a potential survival benefit with adjuvant RT. In a Surveillance, Epidemiology, and End Results (SEER) database analysis of 3,839 patients with iCCA, 273 patients (7%) received surgery followed by adjuvant RT [39]. Median OS was 11 months (95% CI 9–13) with adjuvant RT compared with 6 months for surgery alone (*p* = 0.014). On multivariable analysis, surgery plus adjuvant RT was associated with improved OS compared with no treatment, with a hazard ratio of 0.40 (95% CI 0.34–0.47). After propensity adjustment, adjuvant RT remained associated with improved survival compared with surgery alone, with a hazard ratio of 0.82 (95% CI 0.70–0.96). Although intriguing, this SEER analysis cannot in itself justify routine adjuvant RT.

Subsequent registry analyses suggested that any benefit from adjuvant radiation may be concentrated in selected high-risk subgroups. In a Taiwan Cancer Registry study including 599 patients with iCCA, 174 received adjuvant concurrent chemoRT and 146 received adjuvant chemotherapy followed by RT [40]. Among patients with pathologic stage III to IV disease, adjusted hazard ratios for overall mortality were 0.55 (95% CI 0.41–0.74) for concurrent chemoRT and 0.92 (95% CI 0.70–1.33) for chemotherapy followed by radiation compared with chemotherapy alone cohort. In margin positive early-stage disease, concurrent chemoRT was associated with improved survival compared with chemotherapy alone, with an adjusted hazard ratio of 0.65 (95% CI 0.56–0.92). In advanced stage disease, concurrent chemoRT was associated with improved survival in both margin negative and margin positive subsets, with adjusted hazard ratios of 0.71 (95% CI 0.54–0.80) and 0.51 (95% CI 0.36–0.75), respectively.

Single institution series have suggested benefit of adjuvant chemoRT. In a retrospective cohort of 95 patients, including 33 patients with iCCA among which 26% received adjuvant chemoRT [41]. Median OS was 30.2 months with adjuvant chemoRT compared with 26.3 months with observation, although this difference did not reach statistical significance (*p* = 0.0695). On multivariable analysis, adjuvant chemoRT was associated with improved disease-free survival (hazard ratio 0.50, *p* = 0.03) and improved OS (hazard ratio 0.37, *p* = 0.004), including benefit in both margin negative (hazard ratio 0.34, *p* = 0.035) and margin positive (hazard ratio 0.15, *p* = 0.003) subsets.

Meta-analyses across biliary tract cancers provide additional context but offer limited evidence specific to iCCA. In a pooled analysis of 6,712 patients with biliary tract cancers, adjuvant therapy was associated with improved OS compared with surgery alone, with the greatest benefit observed in node positive and R1 disease [42]. However, only one study in this analysis included patients with iCCA, limiting applicability to this subgroup. A 2020 meta-analysis of 22 studies including 10,181 iCCA patients similarly reported an OS benefit with adjuvant therapy (hazard ratio 0.63, 95% CI 0.52–0.74) [43]. Chemotherapy demonstrated the strongest association with improved survival (hazard ratio 0.57, 95% CI 0.44–0.70), whereas RT alone showed a weaker and non-significant effect (hazard ratio 0.71, 95% CI 0.39–1.03). Combined chemoRT was associated with a modest survival benefit (hazard ratio 0.73, 95% CI 0.57–0.89).

In contrast, more contemporary large database analyses focused specifically on resected iCCA have challenged the benefit of adjuvant RT. A propensity matched National Cancer Database (NCDB) analysis of more than 5,000 patients treated between 2006 and 2018 found no OS benefit for adjuvant RT, with or without chemotherapy, compared with chemotherapy alone or observation, including in patients with positive margins [44]. Similarly, an NCDB analysis of 20,005 patients with non-metastatic iCCA reported the longest median OS with surgery alone at 46.3 months, with no significant improvement when surgery was combined with chemotherapy (46.0 months, *p* = 0.10) or chemoRT (44.5 months, *p* = 0.11) [45]. These findings have been reported in abstract form only, and further sub-analyses incorporating resection margin status and lymph node involvement may better clarify whether selected high-risk subsets derive benefit from adjuvant chemoRT.

Current guideline recommendations reflect this uncertainty. NCCN lists adjuvant systemic therapy as the preferred approach following resection, with chemoRT considered an option but not preferred in higher risk settings [46]. The American Society of Clinical Oncology does not recommend routine use of adjuvant radiation for iCCA, emphasizing the lack of a well-defined postoperative target and the high competing risk of distant failure [47].

## External Beam Radiation Therapy in Metastatic or Palliative Settings

Even in the setting of metastatic iCCA, progressive liver involvement and tumor related hepatic failure remain major drivers of morbidity and mortality. This observation has motivated interest in liver directed radiotherapy for selected patients with liver dominant disease and controlled extrahepatic burden, with the goal of prolonging survival and mitigating liver related death.

Retrospective data suggest a potential survival benefit from local liver RT in this context. In a study evaluating iCCA patients with metastatic extrahepatic disease at diagnosis who achieved stable disease or better disease following chemotherapy, receipt of liver directed RT was associated with improved OS compared with chemotherapy alone [48]. Among 61 patients treated with liver RT and 220 treated with chemotherapy alone, median OS was 21 months (95% CI 17–26) versus 9 months (95% CI 8–11), respectively. Tumor related liver failure accounted for death more frequently in the chemotherapy alone group than in the liver RT group (82% versus 47%, *p* = 0.001). The median BED delivered to the liver was 97.5 Gy (IQR 80.5–97.9). On multivariable propensity score matched analysis, longer duration of upfront chemotherapy was associated with reduced mortality (HR 0.82, *p* = 0.005), while receipt of liver RT was independently associated with improved survival (HR 0.40, *p* = 0.002).

Additional institutional series provide further evidence supporting SBRT in advanced disease. In a retrospective cohort of 17 patients with advanced or metastatic iCCA treated with SBRT, median OS from diagnosis was 21 months (95% CI 14.5–27.4) [49]. OS at 1-year and 2-years was 90% and 30%, respectively. LC was high, with rates of 92% at 1-year and 70% at 2-years, whereas PFS was lower, at 35% at 1-year and 15% at 2-years. SBRT was delivered to doses of 30 to 50 Gy over 5 to 10 fractions, and no cases of SBRT related cholangitis or liver failure were observed.

Population based analyses further support a role for liver directed RT in selected metastatic patients. An NCDB study of 2,201 patients with metastatic iCCA treated with chemotherapy with or without hepatic surgery or EBRT of at least 45 Gy demonstrated improved OS with liver directed RT [50]. On multivariable analysis, the hazard ratio for death was 0.60 (95% CI 0.48–0.74; *p* < 0.001). In a propensity matched comparison of 208 patients receiving chemotherapy alone versus 104 receiving chemotherapy plus liver directed RT, the association persisted with a hazard ratio of 0.57 (95% CI 0.44–0.74; *p* < 0.001). Survival benefit remained consistent in landmark analyses at 3 months (HR 0.61, 95% CI 0.47–0.79; *p* < 0.001), 6 months (HR 0.68, 95% CI 0.50–0.92; *p* = 0.01), and 12 months (HR 0.68, 95% CI 0.47–0.98; *p* = 0.04).

Beyond disease control, EBRT continues to play an important role in symptom palliation. Conventional fractionation EBRT has been shown to provide meaningful relief of pain and jaundice in patients with unresectable disease. In a retrospective study of 84 patients with unresectable iCCA, EBRT delivered to a median dose of 50 Gy (range 30–60 Gy, 1.8–2.0 Gy per fraction) was associated with improved median OS compared with no EBRT (9.5 versus 5.1 months, *p* = 0.003), with 1-year OS rates of 38.5% versus 16.4%, respectively [51]. Among 19 patients presenting with jaundice, complete relief was achieved in 36.8% and partial relief in an additional 31.6%. Similarly, another retrospective series of 75 patients reported higher 1-year OS with EBRT compared with no EBRT (36.1% versus 19.0%, *p* = 0.021) and pain relief in 90% of patients receiving EBRT, using a median total dose of 50 Gy delivered in 2 Gy fractions [52].

More recently, randomized data have supported the role of EBRT for symptom palliation in liver malignancies. A phase III trial demonstrated that single fraction whole liver RT of 8 Gy provided clinically meaningful improvement in patient reported pain in a cohort including multiple hepatic tumor types, supporting the use of EBRT for symptom control even in advanced disease settings[53].

## External Beam Radiation Therapy Combined with Immunotherapy

The rationale for combining RT with immunotherapy is supported by evidence that SBRT can modulate the tumor microenvironment, enhance antigen presentation, and promote antitumor immune responses, potentially synergizing with immune checkpoint inhibition [54, 55]. In iCCA, clinical evidence for this strategy remains early and is largely limited to small prospective studies, retrospective series, and case reports.

Prospective clinical data are emerging. A phase II study evaluated RT followed by anti PD-1 therapy with camrelizumab in 36 patients with unresectable primary or postoperative recurrent iCCA without distant metastases [56]. RT was delivered to a dose of at least 45 Gy in 2 to 2.5 Gy per fraction. Reported outcomes included a 1-year PFS rate of 44.4% (95% CI 30.8–64.0), median PFS of 12.0 months (95% CI 7.5–not estimable), and median OS of 22.0 months (95% CI 15.0–not estimable). The objective response rate was 61.1%, and the disease control rate was 86.1%. These results compare favorably to historical chemotherapy-only outcomes. Grade 3 or higher treatment related adverse events occurred in 14% of patients, most commonly lymphopenia (5.6%), with additional events including bullous dermatitis (2.8%), thrombocytopenia (2.8%), and deep vein thrombosis (2.8%).

Case based evidence suggests that RT may sensitize tumors to immunotherapy even in biological contexts traditionally associated with limited checkpoint inhibitor activity. One case series described three patients with advanced or recurrent iCCA, including one patient with stage IVA disease and two with postoperative recurrence, all characterized by low tumor mutational burden, microsatellite stable status, and negative PD-L1 expression [57]. These patients were treated with SBRT delivered to 52 Gy in 4 fractions or 55 Gy in 5 fractions in combination with pembrolizumab or nivolumab, with reported responses in both irradiated and non-irradiated lesions, consistent with an abscopal effect. A separate case report described a patient with metastatic iCCA treated with pembrolizumab and SBRT to the liver (50 Gy) and lung (48 Gy), achieving complete response and ongoing survival at 26 months follow up after treatment, despite low PD-L1 expression, in the setting of microsatellite instability high disease [58].

Additional reports have described disease control and potential conversion to resectability with combined RT and immunotherapy. In a series of four patients with refractory advanced cholangiocarcinoma treated with anti PD-1 therapy administered concurrently with or following SBRT, disease control by RECIST criteria was observed in all cases, with conversion to operability in one initially unresectable patient [59].

Building on these early signals, ongoing prospective studies are evaluating the integration of SBRT with immune checkpoint inhibition, including a phase II trial investigating nivolumab combined with SBRT following induction chemotherapy in cholangiocarcinoma [60].

Overall, while early clinical signals suggest potential synergy between EBRT and immunotherapy in iCCA, current evidence remains preliminary. Prospective trials are needed to define optimal sequencing, patient selection, radiation dose and fractionation, and safety of combined modality approaches.

## Conclusion

EBRT has become an increasingly important component of multidisciplinary management for iCCA because it is broadly applicable across tumor sizes and locations, does not depend on tumor vascularity, and can be delivered with modern conformal techniques that enable meaningful dose escalation (Table 1). The most consistent evidence signal across definitive series is a dose response relationship, with higher BED and ablative intent strategies associated with improved LC and OS in unresectable disease [14, 15, 61]. Advanced technology such as proton therapy and MRgRT can potentially expand the delivery of ablative dose of RT for selected patients, particularly when gastrointestinal proximity constrains photon based SBRT [21–25]. Beyond definitive therapy, EBRT contributes to downstaging strategies that can convert a subset of initially unresectable patients to resection with favorable long-term outcomes [26, 27]. In contrast, the postoperative role of adjuvant RT remains controversial in iCCA with conflicting retrospective evidence and guideline caution emphasizing systemic therapy as preferred [32, 44, 47]. In metastatic settings, emerging retrospective and database evidence suggests that liver directed RT may improve survival in carefully selected patients with liver dominant disease and may reduce deaths due to tumor related liver failure, while palliative EBRT remains valuable for symptom control including pain and jaundice relief [48–52]. Finally, early phase studies and case reports of EBRT combined with checkpoint inhibitors suggest promising activity in selected patients, warranting prospective validation to define the optimal integration of RT within evolving systemic treatment paradigms for iCCA [56, 60].

**Table 1:** Clinical contexts for the use of EBRT in iCCA

Clinical setting and intent	Typical patient candidates and goal of treatment	EBRT approach and key technical considerations	Relevant references
Definitive EBRT for unresectable iCCA	Unresectable, liver dominant disease, goal is durable local control and prevention or delay liver failure	Image guided RT delivered in 3 to 5 fractions (SBRT), 10 to 15 fractions (hypofractionation) or 25 to 35 fractions (conventional), incorporating respiratory motion management and sparing of normal liver and nearby gastrointestinal organs	Sebastian et al., [8]; Lee et al., [12]; Smart et al., [14]; Tao et al., [15]; Lee at al, [16], Crane et al., [17]; Liu et al., [18]
Definitive EBRT using PBT	Unresectable large or centrally located tumors with limited uninvolved liver reserve, goal is to reduce radiation exposure to normal liver and facilitate delivery of ablative dose of radiation	Proton therapy planning designed to limit dose to uninvolved liver, with attention to respiratory motion and tumor position relative to stomach or bowel	Yamazaki et al., [21]; Hong et al., [22]; Parzen et al., [23]
Definitive EBRT with MR guided adaptive RT	Unresectable tumors near stomach or bowel, limited CT visibility, or substantial day to day bowel variation, goal is to maintain target coverage while meeting daily normal tissue constraints	MR guided treatment with real time visualization and adaptive replanning to accommodate daily anatomic changes and respiratory motion	Henke et al., [24]; Rosenberg et al., [25]
EBRT for tumor downstaging and conversion to resection	Initially unresectable or borderline resectable disease with surgical potential, goal is to enable complete resection	EBRT, often delivered with concurrent systemic therapy, with reassessment of resectability after treatment response	Cho et al., [26]; Sumiyoshi et al., [27]
EBRT as a bridge to liver transplantation	Highly selected patients in protocol based frameworks or clinical trials, goal is to maintain disease control while awaiting transplant	EBRT incorporated into neoadjuvant or bridging strategies with close coordination of timing with transplant evaluation and systemic therapy	Wu et al., 2024[37]
Adjuvant EBRT after surgical resection	Selected high risk features such as positive margins or node positive disease, goal is to reduce risk of locoregional recurrence	Postoperative EBRT, often with concurrent chemotherapy, with careful definition of the tumor bed and regional lymphatic targets	Shinohara et al., [39]; Lin et al., [40]; Dover et al., 2016[41]; Horgan et al., [42]; Ke et al., [43]; Seo et al., [44]; Mohsin et al., [45]
EBRT for metastatic disease or for palliation	Patients with liver dominant metastatic disease or symptoms attributable to tumor burden such as pain, biliary obstruction related symptoms, bleeding, or mass effect, goal is to improve disease control and or relieve symptoms	EBRT to liver lesions and or symptomatic metastatic sites, typically delivered in abbreviated courses, often 1 to 10 fractions depending on clinical scenario	De et al., [53]; Sharma et al., [49]; Sebastian et al., [50]; Chen et al., [51]; Zeng et al., [52]; Dawson et al., [53]

*EBRT* External Beam Radiation Therapy, *iCCA* Intrahepatic Cholangiocarcinoma, *PBT* proton beam therapy, *SBRT* Stereotactic Body Radiotherapy

## Data Availability

No datasets were generated or analysed during the current study.
